# The Predictive Validity of the SAPROF-SO for Success on Supervised Release From a Secure Treatment Center

**DOI:** 10.1177/10790632251328958

**Published:** 2025-04-13

**Authors:** Angela E. Carr, Sharon M. Kelley, Gwenda M. Willis, David Thornton

**Affiliations:** 1 1415 University of Auckland, Auckland, New Zealand; 2 144159 Sand Ridge Secure Treatment Center, Madison WI, USA; 3 Forensic Assessment, Training, & Research (FAsTR), LLC, Madison, WI, USA

**Keywords:** sexual offending, recidivism, SAPROF-SO, protective factors, assessment

## Abstract

Recent research has highlighted the importance of protective factors in preventing sexual offense recidivism and led to the development of a number of strengths-based approaches to the treatment of adult males who have been convicted of sexual offenses. However, these approaches have not been supported by structured methods to assess protective factors. The Structured Assessment of PROtective Factors against Sexual Offending (SAPROF-SO) was designed to bridge the gap between assessment and strengths-based treatment, and the current study contributes to the validation of that instrument. The SAPROF-SO was rated retrospectively for adult males released on supervised release from a secure treatment center in the US (*N* = 170). SAPROF-SO total scores predicted supervised release success as measured by revocation outcomes. In addition, results highlighted the utility of the SAPROF-SO Resilience subscale in predicting supervised release revocation for any reason and the Adaptive Sexuality subscale in predicting sexually related revocations. Notably, the SAPROF-SO demonstrated incremental validity over the Static-99R, which was not predictive of revocation outcomes. Implications for the management of sexual offense risk when planning and administering release from criminal justice contexts are discussed.

## Introduction

Given the incalculable social, psychological and financial costs of sexual crimes, their prevention is of critical importance. Further, it is now well established that one of the most robust predictors of future sexual offending, is having a past history of committing sexual offenses (Hanson & Morton-Bourgon, 2005). However, research has also shown that when delivered according to the principles of the “Risk-Need-Responsivity Model for Offender Assessment and Rehabilitation” (RNR; see Bonta & Andrews, 2017), sexual offense treatment programs can significantly decrease sexual offense recidivism (e.g., Hanson et al., 2009).

The three core principles of the RNR model specify that: (1) the level of treatment provided to an individual should match their risk of re-offending, (2) treatment should directly target the modifiable beliefs, behaviors, skills and social factors that increase an individual’s risk of recidivism (criminogenic needs), and (3) the likelihood of treatment effectiveness will be maximized if the intervention is effectively tailored to be responsive to these individuals’ learning styles, motivation, abilities and strengths (Bonta & Andrews, 2007). Of course, to address these principles, one must first be able to effectively assess recidivism risk, criminogenic needs, and identify relevant responsivity factors.

### Assessing Recidivism Risk

Since the late 1990s, a substantive body of research has focused on developing actuarial tools for effectively assessing sexual recidivism risk. Initially, this involved identifying personal and criminal history correlates of recidivist sexual offending and quantifying these into instruments such as the RRASOR (Hanson, 1997), SACJ-Min (Grubin, 1998), Static-99 (Hanson & Thornton, 1999), Static-99R (Helmus et al., 2012), Static-2002 (Hanson & Thornton, 2003), Static-2002R (Helmus et al., 2012) Risk Matrix, 2000 (Thornton, 2007). As stated by Bonta and Andrews (2007), use of these structured instruments enabled significantly increased risk assessment reliability compared to clinical judgement alone, and many remain in common use today. Indeed, according to Helmus et al. (2022, p. 307), the Static-99R is currently the “most commonly used and researched risk assessment tool for individuals charged or convicted of sexual offenses”. However, a significant limitation of these instruments is that the factors they measure tend to be static in nature (Coid et al., 2016). That is, they are either unlikely to change, or only likely to change in one direction (e.g., increase). As such, they do not account for changes in an individual’s criminogenic needs that could increase or reduce their recidivism risk.

Over the last two decades research has increasingly focused on accounting for these more changeable (or “dynamic”) factors in the assessment of sexual offense recidivism risk. This has led to the development of instruments such as the Violence Risk Scale - Sexual Offense Version (VRS-SO; Olver et al., 2007), STABLE-2007 (Hanson et al., 2007) and ACUTE-2007 (Hanson et al., 2007). These instruments include measures of attitudinal, behavioral and social factors that have been found to discriminate individuals who commit recidivist sexual offenses from those who don’t, after controlling for static risk assessment scores (Brankley et al., 2021; Hanson & Harris, 1998, 2000; Olver et al., 2007). Compared to those that measure static risk, measures of dynamic risk generally require more training and information to administer but, in addition to incrementally adding to the validity of individual risk assessment, they are also helpful in identifying treatment targets (Hogan & Sribney, 2019). As such, their use can enhance treatment program’s ability to meet the second RNR principle.

Despite the aforementioned gains, research has consistently shown that up to half of adult males who have been convicted of sexual offenses and assessed as having both high static and dynamic risk do not sexually re-offend, even when followed up for more than 20 years (e.g., Hanson et al., 2018, 2022; Lussier et al., 2023; Olver et al., 2018). Indeed, as shown by Hanson et al. (2014), those who do sexually reoffend are most likely to do so during the first five years following release. In turn, they recommended the need for further research “to explain the substantial rate of desistance by high-risk sexual offenders” (p. 2792). Subsequent research into offense desistance suggests that the presence of so called ‘protective factors’ may explain the difference between adult males who are assessed as being of high or very high risk and who go on to reoffend, and those assessed at the same level but who do not reoffend (Klepfisz et al., 2017).

### From Risk to Protection

Protective factors can be defined as factors that decrease the likelihood of a negative outcome happening. In terms of sexual offense recidivism, they might directly reduce re-offense risk irrespective of risk factors, and/or moderate the influence of risk factors (de Vogel et al., 2009; de Vries Robbé et al., 2015; Willis et al., 2017–2020). Many sexual offense protective factors exist on a continuum with risk factors. However, just because a risk factor is absent, does not mean that its corresponding protective factor is present. For example, evidence has shown that involvement in pro-offending social networks increases recidivism risk (Mann et al., 2010a) and involvement in prosocial networks protects against it (Willis & Grace, 2008, 2009), but just because someone is not involved with pro-offending social networks does not mean they have prosocial associates (or indeed any associates at all).

Recognition of the importance of protective factors in preventing future sexual offenses has led to the development of a number of strengths-based approaches to the treatment of adult males who have been convicted of sexual offenses (e.g., Laws & Ward, 2011; Marshall et al., 2017). However, these developments have not been supported by the availability of structured methods to assess and monitor the protective factors needs of those adult males. Consequently, professionals working with adult males who have been convicted of sexual offenses are limited in terms of their ability to evaluate the effectiveness of treatment interventions that target protective factors and/or feed the results into judicial decisions (such as readiness for discharge or parole). Further, given that recidivism risk assessments commonly inform forensic and correctional setting decisions regarding the form and intensity of treatment and supervision, this limitation may also be impacting associated professionals’ ability to effectively tailor treatment and supervision to the specific needs of the adult males they are working with.

In response to these limitations, some validated risk assessment instruments, such as the Dynamic Risk Assessment scale for Offender Reentry (DRAOR; Serin et al., 2007, 2010), now include subscales for assessing protective factors amongst individuals who have been convicted of crime generally, and one – the Structured Assessment of PROtective Factors for violence risk (SAPROF; de Vogel et al., 2009) – provides a comprehensive measure of protective factors against violence in adults. However, until recently, none have been specifically designed to measure protective factors against sexual reoffending (Willis et al., 2020). In the same way that some risk factors are unique to sexual offending (vs. general or violent offending; van der Put et al., 2020), research and clinical observations suggest that some protective factors may uniquely protect against sexual offending (e.g., prosocial sexual interests; de Vries Robbé et al., 2015; Yoon et al., 2018).

### The SAPROF-SO

In response to the dearth of structured protective factors assessment instruments for specific use with individuals who have been convicted of sexual offenses, Willis et al. (2021) developed the Structured Assessment of Protective Factors against Sexual Offending (SAPROF-SO). Initially derived from the SAPROF, the SAPROF-SO is a new instrument consisting of 14 items across three subscales: *Resilience, Adaptive Sexuality, and Prosocial Connection & Reward* and an optional six-item *Professional Risk Management* subscale. Items were informed by the sexual offense desistance literature and the strengths-based *Good Lives Model* (GLM; see Ward & Stewart, 2003), as well as attributes that have been shown by researchers (such as Bonta & Andrews, 2017; Hanson et al., 2007; Mann et al., 2010a) to protect against the manifestation of criminogenic needs (see Table 1 for a full list of items). Use of the SAPROF-SO is underpinned by a detailed coding manual (Willis et al., 2021) and users are also encouraged to complete a certified training program (Kelley et al., 2022).

**Table 1.** SAPROF-SO Descriptive Statistics.

Subscale	Items	*n* valid	Mean	SD
Resilience	Adaptive schemas	170	1.30	1.18
	Empathy	149	1.77	1.57
	Coping	169	1.79	1.20
	Self-control	169	2.12	1.34
	Attitudes towards rules and regulations	170	2.25	1.38
	Resilience average	170	1.84	1.14
Adaptive sexuality	Sexual self-regulation	168	1.39	0.97
	Prosocial sexual interests	169	1.36	0.87
	Prosocial sexual identity	167	1.58	1.74
	Intimate relationship	170	0.13	0.43
	Adaptive sexuality average	170	1.12	0.80
Prosocial connection & reward	Goal-directed living	159	1.52	1.17
	Work	169	1.91	0.63
	Leisure activities	161	1.57	0.80
	Social network	170	2.35	1.12
	Emotional connection to adults	148	1.06	1.21
	Prosocial connection & reward average	170	1.71	0.70
Average SAPROF-SO score		170	1.58	0.74

Preliminary research has provided support for the SAPROF-SO’s interrater reliability and construct validity in both community and inpatient samples (see Willis et al., 2020). In addition, a recent retrospective study by Nolan et al. (2023) provided evidence for its predictive and incremental validity. Their sample comprised of 210 adult males who had been convicted of sexual offenses against children in New Zealand, with a mean Static-99R score corresponding to the Average risk category. Nolan et al. found that, after an average follow-up of 12.24 years, archivally rated SAPROF-SO scores inversely predicted sexual recidivism (AUC = .81). In addition, SAPROF-SO scores were also inversely predictive of violent and general recidivism, albeit to a lesser extent (AUCs = .66 and .63, respectively). Using a series of hierarchical regressions, Nolan et al. demonstrated that the SAPROF-SO maintained its predictive ability after controlling for participants’ Static-99R risk scores.

### The Current Study

The current research aimed to add to the SAPROF-SO validation process by establishing the extent to which the SAPROF-SO could also be used to predict supervised release success in a US context involving individuals identified as being of significantly higher risk (on average) of sexual recidivism than those in earlier samples. To this end, the SAPROF-SO was scored for a sample of adult males who had been convicted of at least one sexual offense, detained under civil commitment legislation at a secure treatment center in the US and then released (under supervision) into the community for a period of at least 24 months. Those scores were then compared statistically to each adult male’s supervised release revocation data. The latter included details of any breaches of supervised release rules (including breaches due to sexual offending and associated behavior).

Another aim of this research was to examine the predictive validity of the SAPROF-SO alongside established risk assessment tools and investigate the extent to which use of the SAPROF-SO could enhance the predictive ability of associated assessment processes (i.e., the incremental predictive validity of the SAPROF-SO) within the sample. In addition, to aid ongoing development and use of the SAPROF-SO, particularly in clinical contexts, the research sought to determine whether any of the SAPROF-SO subscales were more likely than others to predict success on supervised release.

This research was supported by a Rutherford Discovery Fellowship awarded to the third author. Neither the funders, nor the Sand Ridge Secure Treatment Center Research Unit had any involvement in the analysis process and/or preparation of the manuscript. The authors take responsibility for the integrity of the data, the accuracy of the data analyses, and have made every effort to avoid inflating statistically significant results.

## Method

### The Setting

The current research involved adult males who had been civilly committed to Sand Ridge Secure Treatment Center (SRSTC) under the Sexually Violent Persons (SVP) law in the US state of Wisconsin. SRSTC was established in 2001 for the purpose of housing individuals who had been convicted of sexually violent offenses as defined by statute, had been assessed as having a predisposing mental condition that made them likely to commit sexually violent acts in the future, and had been assessed as having a lifetime risk for sexually violent acts that was more likely than not (i.e., a lifetime risk threshold of above 50%). While civilly committed, individuals can either be placed at SRSTC or in the Supervised Release program, which is geared for individuals who have completed earlier stages of treatment at the main facility and are ready to practice learned skills in the community (see Ambroziak et al., 2023 for a review).

Supervised release is highly restrictive. Individuals are subject to ongoing supervision and monitoring (i.e., one-to-one supervision while in the community and GPS monitoring). The statute requires individuals to be monitored at all times whenever they leave their house for the first year of supervised release. Those on supervised release must participate in sexual offense specific treatment and other mental health services as required and are only allowed contact with individuals approved by their supervised release team (and then often only with oversight from team members). Although supervised outings may be permitted for employment or educational purposes, in practice, such activity is rarely obtained until after the first year of supervised release because many clients find it difficult to find employment allowing supervised release monitors to be present. If clients violate the rules of supervised release, their community team may petition the court to return them to SRSTC. Alternatively, if they demonstrate compliance with supervised release rules, they may earn increased freedoms and opportunities.

### Participants

The research aimed to sample all adult males who had been convicted of at least one sexual offense (after the age of 17 years old), civilly committed to SRSTC, and then released into the community (under supervised release conditions) at least 24 months prior to 31 March 2023 (the date at which follow-up data were extracted). Staff at the SRSTC Research Unit were responsible for identifying participants who met these criteria and providing their records to the first author. In total, the records of 177 eligible participants were identified, but three were excluded due to their records containing insufficient data to score the SAPROF-SO (see below). In addition, as we used a fixed follow-up period of 24 months (two years) for all analyses, four were excluded because they had either died within 24 month of supervised release or (in one case) voluntarily requested return to SRSTC. This resulted in a final sample of 170 adult males.

Table 2 provides basic demographic information of the sample, which were most commonly identified as Caucasian (70.0%), and had been convicted of offenses involving female (84.1%), child (70.6%), and victims with whom they were previously acquainted (71.8%). While specific offenses were not coded in the database, 80.6% of the sample had at least two prior sexual offense convictions, 37.1% had four or more sexual offense convictions, and only 4.1% had no prior sexual offense convictions (this data was missing for two cases). Given the statutory requirements for civil commitment in Wisconsin, the entire sample had at least one contact or attempted contact sexual offense that met the legal definition for sexually violent offense (e.g., rape, sexual assault in the 1^st^, 2^nd^, and 3^rd^ degree, etc.). Only 20.6% of the sample had a non-contact sexual offense conviction. The majority had been diagnosed with paraphilic (80.6%) and personality disorders (74.7%).

**Table 2.** Participant Details.

Participant characteristics (*N* = 170)		*n*	%
Ethnic identity	White	119	70.0
	Black	36	21.2
	Native American	10	5.9
	Hispanic origin	5	2.9
Diagnoses	Any paraphilic disorder	137	80.6
	Pedophilic disorder	85	50.0
	Sexual sadism disorder	14	8.2
	Other specified paraphilic disorder (e.g. coercive or hebephilic)	42	24.7
	Any other paraphilic disorder	28	16.5
	Major mental illness	14	8.2
	Alcohol use disorder	67	39.4
	Other substance use disorder	39	22.9
	Any personality disorder	127	74.7
	Antisocial personality disorder	93	54.7
	Any other personality disorder	38	22.4
Convictions	Child victim	120	70.6
	Teen victim	94	55.3
	Adult victim	67	39.4
	Male victim	75	44.1
	Female victim	143	84.1
	Family victim	67	39.4
	Acquaintance victim	122	71.8
	Stranger victim	75	44.1
	Traditional non-contact sexual offenses (e.g., non-image related)	35	20.6
	Enticing a minor using the internet	1	0.6
	Child abuse images	5	2.9
Treatment phase^a^	Motivational (MAP - to help address treatment interfering factors)	11	6.5
	Phase 1	11	6.5
	Phase 2	46	27.1
	Phase 3	97	57.1
	Not in treatment/Withdrawn	5	2.9
Supervised release data	Met criteria for supervised release	102	60.0
	Met criteria for discharge	119	70.0
Static-99R risk categories	III – Average risk (Scores 1–3)	35	20.6
	IVa – Above average risk (Scores 4–5)	73	42.9
	IVb – Well above average risk (Scores 6 and above)	61	35.9

^a^The inpatient SRSTC SVP Treatment Program has 3 phases. Phase 1 focuses on developing self-management skills, Phase 2 emphasizes identification of re-offense risk factors and ways they may manifest in current functioning, and Phase 3 is about managing those risk factors, replacing them with adaptive behaviors and preparing for community transition. The phase model is not rigidly applied, and individuals in more advanced phases being able to work on issues from earlier phases, and those in earlier phases doing more advanced work in preparation for associated transitions. The model also includes a motivational stream (MAP), to help individuals address treatment interfering factors.

Almost all of the participants were engaged in treatment at the time of release (97.1%) but less than two-thirds of them were clinically judged to have made sufficient treatment progress to meet the criteria for supervised release (60.0%). Indeed, more were identified as meeting the criteria for discharge (70.0%). At the time of supervised release, participants’ mean age was 51.8 years old (*SD* = 11 years) and the mean duration of their criminal justice institutionalization to date was 19.6 years (*SD* = 7.1 years).

All reported data were obtained from institutional records and publicly accessible court records. No direct contact with participants was made. This study was approved by the site’s Institutional Review Board (IRB).

### Measures and Data Collection Procedures

#### SAPROF-SO

The SAPROF-SO is an instrument designed to assess protective factors against sexual recidivism. It comprises 14 items that have been empirically or theoretically associated with decreased sexual recidivism risk. These items are grouped into three subscales: *Resilience, Adaptive Sexuality, and Prosocial Connection & Reward* (Willis et al., 2021). The SAPROF-SO is underpinned by a certified training program and a detailed coding manual. Prior to commencing the current research, the first author underwent training in the use of the SAPROF-SO. She then used the coding manual to retrospectively generate SAPROF-SO item scores for each participant, based on information contained in their most recent SRSTC records at the time of supervised release. These records included participants annual SVP Examination Reports and Treatment Progress Reports, as well as information referenced in these reports (e.g., Treatment Progress Summary notes, Social History Update Reports, Treatment Program Polygraph Evaluation Reports and Sexual Fantasy and Masturbation Log Polygraph Summaries). As per SAPROF-SO guidelines, only information pertaining to the relevant time periods (usually the last six or 12 months prior to release) was considered relevant for the purpose of scoring. Scoring was primarily completed by the first author. However, the records of the first 41 participants (24.1%) were also independently scored by either the second or third author and interrater reliability scores were calculated.

Table 1 provides descriptive statistics for the complete SAPROF-SO dataset. Scores for each item ranged from 0, where there was no evidence of the item’s presence, to 4, where the evidence indicated that the item was clearly present. A score of 2 indicated that the item was present “somewhat” (see Willis et al., 2021 for further coding details). As evident in Table 1, the retrospective nature of the scoring process meant that sometimes there was insufficient information available in the records to score a particular item. This was most common for items in the Prosocial Connection & Reward domain, where 12.9% of cases did not include data for Emotional Connection to Adults, 5.3% did not include Leisure Activities data, and 6.5% did not include Goal Directed Living data. In addition, 12.4% of cases did not include data pertaining to one of the Resilience items (Empathy). In these cases, it was recognized that just because the records did not explicitly mention information relevant to coding an item did not mean that it was not present, therefore no scores were entered. If more than one item in the Adaptive Sexuality subscale, or more than two items in any other subscale could not be rated, records were assessed as insufficient, and the participant was excluded from the dataset. In cases missing information for one or two items, the subscales were subsequently prorated (by dividing the total number of items in the subscale by the total rated for the participant and then multiplying the sum by the total participants’ total subscale score).

#### Risk Assessment Tools

As part of the process of preparing SVP Examination Reports, examiners employed at SRSTC usually administer sexual offense recidivism risk assessment instruments, including the Static-99R and VRS-SO. The current study initially aimed to examine the predictive validity of the SAPROF-SO alongside each of these. However, after reviewing the data provided by the SRSTC research unit, we found that of the 170 participants sampled, only 65 (38.2%) had been administered the VRS-SO. Consequently, the planned analyses involving this instrument were abandoned.

##### Static-99R data

The Static-99R (Helmus et al., 2012) is an assessment instrument that measures sexual offense recidivism risk factors that cannot be reduced by behavioral change. It consists of 10 items, relating to individuals’ criminal history, demographic information, and victim characteristics. Resulting scores range between −3 and 12, with those below −1 indicating “very low” risk, those between −1 and 0 indicating “below average risk”, those between 1 and 3 indicating “average risk”, those between 4 and 5 indicating “above average risk” and those above 5 indicating “well above average risk” (Hanson et al., 2017). Importantly, the current Static-99R is a revised version of the original Static-99 and includes updated age weights which have reportedly enhanced its ability to account for the now well-established relationship between increasing age and declining sexual offense recidivism (Helmus et al., 2012).

For the current study, Static-99R scores were obtained from participant records and provided to the first author by SRSTC Research Unit staff at the same time as they provided the revocation data (after SAPROF-SO coding had been completed). The vast majority of participants’ Static-99R scores fell in the above average or well-above average risk categories (*M* = 4.98, *SD* = 1.62, see Table 2).

#### Supervised Release Revocation Data

Once the SAPROF-SO was scored for each participant, SRSTC Research Unit staff provided the first author with associated revocation data. Importantly, this process meant that the SAPROF-SO scoring process was “blinded” such that the first author was not aware of participant outcomes until after she had assessed each participant against the SAPROF-SO item criteria and allocated an associated score.

The revocation data included details of any breach (es) of supervised release rules (including sexual offense-related breaches and sexual offending) that resulted in a return to custody at SRSTC. In total, 68 participants (40.0%) were revoked within 24 months of supervised release from SRSTC (Table 3). All but five participants (98.0%) were revoked for more than one violation (*M* = 4.72, *SD* = 2.38). Notably, all of those who were identified as having committed a sexual offense had also violated other supervised release rules. In addition to those who were identified as having committed sexual offenses whilst on supervised release, 20 participants (11.8%) were revoked for behaviors that did not meet the legal criteria of a sexual criminal offense, but reportedly demonstrated re-activation of interests and/or behaviors associated with their prior sexual offending patterns (e.g., offense-related interests apparently activated with no imminent risk, engaged in behavior which creates opportunity for grooming of victims/victim-protectors). In two of these cases, that behavior was the only rule violation recorded. Given that researchers have identified such offense analogous behaviors as possible proxy measures of sexual offending in highly restrictive contexts (such as SRSTC supervised release) where the opportunity to actual sexual offending is very limited (Mann et al., 2010b; Pearson & McDougall, 2017), it was determined that the current study would also consider the relationship between SAPROF-SO scores and these outcomes.

**Table 3.** Sample (*N* = 170) Revocation Outcomes During a Fixed Follow-Up Period of 24 Months.

Custody event/revocation	Frequency	%
Any breach of supervised release conditions	68	40.0
• Sexual offense related breach of supervised release conditions (including sexual offenses and sexual offense analogous behaviors)	31	18.2
• Sexual offense	11	6.5
• Any criminal offense (including sexual)	17	10.0

### Planned Analyses

Two-way mixed, absolute agreement single-rater intra-class correlation coefficients (ICCs) were calculated to assess the interrater reliability of SAPROF-SO scores across the subsample of participant files that were independently coded by two raters (*n* = 41). Receiver Operating Characteristics (ROC) analyses were then carried out to assess the predictive validity of the SAPROF-SO and the Static-99R for supervised release revocations. Specifically, these analyses were used to determine whether the SAPROF-SO (total prorated and subscales) and Static-99R scores statistically discriminated between participants who were successful on supervised release (i.e., not revoked) from those who experienced revocations (including revocations for sexual offending, any criminal offending and behaviors that either involved sexual offending, or demonstrated re-activation of interests and/or behaviors associated with prior sexual offending).

One of the advantages of ROC analysis is that it is less likely to be affected by low sexual recidivism base rates than other statistical analysis procedures. AUC values range from 0 to 1, with values closer to 1 or 0 indicating higher levels of discriminant validity while values close to .50 indicate weaker discrimination (Zhou et al., 2002). According to convention, a ROC AUC value of .56 indicates a small effect size, a value of .64 indicates a moderate effect size, and a value of .71 indicates a large effect size (Rice & Harris, 2005). As the SAPROF-SO is designed to measure factors that protect against recidivism, the positive value of the AUC state variable (what the instrument was predicting) was set as 0 (for “no revocation event”) during associated analyses. In turn, because the Static-99R is designed to measure risk of recidivism, its AUC state variable was set as 1 (for “revocation event”). In this way, we were able to ensure that the values specified in associated analyses were consistent (e.g., the closer to 1 the AUC value was, the better the instrument was at predicting the event it was designed to measure).

Finally, a series of hierarchical Logistic Regression Analyses were undertaken to investigate the extent to which the SAPROF-SO and its subscales contributed incremental validity in predicting each type of revocation (any, sexually related, sexual offenses and any offenses) after controlling for participants’ Static-99R scores. The Static-99R scores were entered as Step 1 of each model, with either the SAPROF-SO Total score or the Resilience, Adaptive Sexuality and Prosocial Connection & Reward Subscale scores entered as Step 2. This resulted in the generation of 16 separate models. Logistic Regression Analyses were chosen because they enable direct comparison of the strength of associations between various predictors and outcomes when the latter is binary in nature. They also have the advantage of being relatively resilient to outliers, compared to other regression models. Two measures of model fit are reported. Larger Nagelkerke *R*^2^ values range from 0 to 1, with those closer to 1 indicating that the model is more predictive. Hosmer-Lemeshow test results assess deviation from the model with statistically significant deviation potentially arising if non-linear effects are present or if the logistic link function was inappropriate.

## Results

### Interrater Reliability

The single-rater ICC for the total of the three SAPROF-SO subscales was 0.91, 95% CI = [.84, .95], for the Resilience subscale it was 0.95, 95% CI = [.89, .97], for the Adaptive Sexuality subscale it was 0.65, 95% CI = [.43, .80] and for the Prosocial Connection subscale it was 0.77, 95% CI = [.60, .87]. Based on Koo and Li’s (2016) guideline of selecting and reporting intraclass correlation coefficients, this suggests excellent overall and Resilience subscale items interrater reliability, good Prosocial Connection subscale items interrater reliability and moderate Adaptive Sexuality subscale items interrater reliability.

### SAPROF-SO Descriptive Statistics

The mean SAPROF-SO score across all participants was 1.58 (SD = .74) out of a total possible score of 4, with higher total scores indicating higher levels of protective factors (see Table 1). Notably, limited variability was observed across some items, particularly Intimate Relationship (Adaptive Sexuality subscale) and Work (Prosocial Connection & Reward subscale). This was likely influenced by the setting in which the data were collected, with participants being unable to work more than a limited number of hours each week and being prevented from maintaining the types of intimate relationships needed to meet the “clearly present” criteria of the SAPROF-SO. Table 4 lists the mean SAPROF-SO item, subscale, and total scores, along with the mean Static-99R scores, grouped by revocation outcomes. Independent samples *t*-test found statistically significant differences. Participants who were revoked for any reason had significantly lower SAPROF-SO total scores (*t* (168) = 2.04, *p* = .021), Resilience subscale scores (*t* (168) = 2.23, *p* = .013), Coping (*t* (167) = 2.35, *p* = .010), Self-Control (t (167) = 2.55, *p* = .006) and Emotional Connection to Adults (*t* (134.082) = 2.59, *p* = .005) scores, compared to those who were not revoked. In addition, participants with sexually related revocations had significantly lower scores on the Adaptive Sexuality subscale scores (*t* (57.570) = 3.25, *p* = .001), Prosocial Sexual Interests (*t* (52.117) = 2.41, *p* = .010), Prosocial Sexual Identity (*t* (53.985) = 3.24, *p* = .001), and Adaptive Schema (*t* (54.316) = 2.45, *p* = .009), compared to those who did not experience sexually related revocations. Finally, participants revoked for any criminal offense had significantly lower Self-Control item scores (*t* (167) = 2.15, *p* = .017) than those who were not revoked for a criminal offense. No statistically significant differences were identified between the Static-99R scores of those who were revoked and those who were not, for any type of revocation.

**Table 4.** Mean (and Standard Deviation) of SAPROF-SO Total, Subscale and Item Scores and Static-99R Scores by Revocations.

Scale	Any revocation (*n* = 68/170)		Sexually related revocations (*n* = 31/170)		Sexual offenses (*n* = 11/170)		Any criminal offenses (including sexual) (*n* = 17/170)	
	.00	1.00	.00	1.00	.00	1.00	.00	1.00
Resilience subscale	2.00 (1.18)*	1.61 (1.04)	1.91 (1.16)	1.52 (.97)	1.86 (1.14)	1.52 (1.13)	1.87 (1.14)	1.57 (1.06)
Adaptive schema	1.42 (1.25)	1.12 (1.04)	1.39 (1.21)**	.90 (.94)	1.31 (1.18)	1.09 (1.22)	1.32 (1.19)	1.12 (1.05)
Empathy	1.89 (1.59)	1.58 (1.53)	1.81 (1.59)	1.56 (1.47)	1.81 (1.59)	1.20 (1.23)	1.79 (1.59)	1.63 (1.45)
Coping	1.96 (1.23)*	1.52 (1.11)	1.86 (1.24)	1.47 (.97)	1.83 (1.20)	1.18 (1.08)	1.84 (1.21)	1.29 (1.05)
Self-control	2.33 (1.40)**	1.81 (1.18)	2.20 (1.37)	1.77 (1.10)	2.16 (1.33)	1.55 (1.29)	2.20 (1.34)*	1.47 (1.12)
Attitude to rules	2.39 (1.37)	2.04 (1.39)	2.30 (1.38)	2.03 (1.43)	2.24 (1.38)	2.45 (1.57)	2.25 (1.39)	2.29 (1.40)
Adaptive sexuality subscale	1.17 (.79)	1.02 (.81)	1.18 (.82)**	.77 (1.05)	1.13 (.81)	.80 (.56)	1.14 (.81)	.84 (.66)
Sexual self-regulation	1.49 (.98)	1.25 (.96)	1.45 (1.00)	1.13 (.81)	1.41 (.98)	1.09 (.83)	1.42 (.98)	1.18 (.88)
Prosocial sexual interests	1.39 (.87)	1.31 (.87)	1.43 (.89)*	1.06 (.73)	1.37 (.88)	1.18 (.75)	1.39 (.88)	1.12 (.78)
Prosocial sexual identity	1.67 (1.75)	1.45 (1.73)	1.76 (1.76)**	0.81 (1.40)	1.63 (1.75)	.91 (1.45)	1.64 (1.74)	1.06 (1.64)
Intimate relationship	.13 (.41)	.13 (.45)	.14 (.46)	.06 (.25)	0.14 (.44)	.00 (.00)	.14 (.45)	.00 (.00)
Prosocial connection & reward subscale	1.76 (.70)	1.62 (.69)	1.71 (.73)	1.69 (.57)	1.70 (.70)	1.77 (.69)	1.71 (.70)	1.68 (.69)
Goal-directed living	1.61 (1.17)	1.39 (1.16)	1.58 (1.19)	1.26 (1.06)	1.53 (1.17)	1.44 (1.13)	1.54 (1.18)	1.33 (1.11)
Work	1.94 (.61)	1.87 (.64)	1.92 (.63)	1.87 (.62)	1.92 (.62)	1.73 (.65)	1.93 (.61)	1.76 (.75)
Leisure activities	1.56 (.83)	1.58 (.75)	1.53 (.84)	1.76 (.51)	1.56 (.81)	1.80 (.42)	1.56 (.82)	1.69 (.60)
Social network	2.39 (1.12)	2.29 (1.12)	2.34 (1.17)	2.42 (0.85)	2.35 (1.11)	2.36 (1.29)	2.37 (1.09)	2.18 (1.33)
Emotional connection to adults	1.25 (1.26)**	.75 (1.06)	1.12 (1.22)	.74 (1.10)	1.06 (1.20)	1.00 (1.41)	1.07 (1.21)	1.00 (1.21)
Average SAPROF-SO score	1.67 (.76)*	1.44 (.70)	1.63 (.77)	1.35 (.57)	1.59 (.74)	1.38 (.68)	1.60 (.75)	1.38 (.64)
Average Static-99R score	4.96 (1.62)	5.00 (1.61)	5.01 (1.60)	4.81 (1.66)	4.98 (1.63)	4.91 (1.30)	4.97 (1.65)	5.06 (1.25)

**p < .01, *p < .05.

### Predictive Validity

Table 5 provides the results of the ROC analyses assessing the ability of the SAPROF-SO subscale and total scores to predict supervised release success. SAPROF-SO total scores were predictive of participants succeeding on supervised release (e.g., not being revoked for any reason) (AUC .595, *p* = .030), and also discriminated participants who were revoked for sexually related breaches of supervised release rules from those who were not revoked (AUC .605, *p* = .033). In addition, the SAPROF-SO Resilience subscale independently predicted the lack of any revocation (AUC .598, *p* = .024), while the Adaptive Sexuality subscale predicted a lack of revocations for both sexual offenses (AUC .622, *p* = .049) and sexually related behavior that either constituted an offense or suggested re-activation of offense related interests (AUC .646, *p* = .002). These effects ranged from small to moderate. In contrast, the Static-99R total scores were not predictive of any types of revocations (AUCs ranging from .463 to .520).

**Table 5.** AUCs for SAPROF-SO Total and Subscale and Total Scores by Revocation Outcomes.

Scale	Any revocation (*n* = 68)		Sexually related revocations (*n* = 31)		Sexual offenses (*n* = 11)		Any criminal offenses (including sexual) (*n* = 17)	
	AUC	95% CI	AUC	95% CI	AUC	95% CI	AUC	95% CI
SAPROF-SO	.595*	[.509, .681]	.605*	[.509, .702]	.563	[.402, .723]	.566	[.440, .692]
Resilience subscale	.598*	[.513, .684]	.594	[.492, .696]	.587	[.414, .760]	.575	[.439, .711]
Adaptive sexuality subscale	.553	[.464, .642]	.646**	[.554, .737]	.622*	[.501, .744]	.605	[.483, .727]
Prosocial connection & reward subscale	.555	[.467, .643]	.517	[.412, .622]	.456	[.287, .625]	.502	[.361, .644]
Static-99R	.498	[.408, .587]	.463	[.352, .575]	.490	[.339, .640]	.520	[.393, .646]

**p < .01, *p < .05.

The protective factors that best predicted success on supervised release were self-control (any revocation AUC .631, *p* = .015, sexual offense AUC .773, *p* < .001, any offense AUC .732, *p* < .001), coping (sexual offense AUC .730, *p* = .043, any offense .668, *p* = .049) and prosocial sexual identity (sexual offense related revocation AUC .638, *p* = .034).

### Incremental Validity

As shown in Table 6, the results of our hierarchical logistic regression analyses suggested that, after controlling for Static-99R scores, SAPROF-SO scores consistently increased the amount of variation in revocation outcomes explained by each model (as indicated by Nagelkerke *R*^*2*^ values). Further, none of the models demonstrated statistically significant predictive value after the Static-99R scores had been entered but, following addition of the Adaptive Sexuality subscale at Step 2 of the sexually related revocation model, significance was achieved (*χ2* (2, *N* = 170) = 7.200, *p* = .027). In this case, the model correctly classified 81.7% of cases and explained 4.2 to 6.8% of the variance in revocation outcomes.

**Table 6.** Hierarchical Logistical Regression Predicting Likelihood of Revocation for Total Sample (*N* = 170) During the 24 Month Follow-Up Period.

		Any revocation			Sexually related revocation			Sexual offense revocation			Any offense revocation		
Scale		Wald (*df* = 1)	Exp(B)	95% CI	Wald (*df* = 1)	Exp(B)	95% CI	Wald (*df* = 1)	Exp(B)	95% CI	Wald (*df* = 1)	Exp(B)	95% CI
Block 1	Static-99R	.024	1.015	[.838, 1.230]	.424	.922	[.723, 1.177]	.021	.972	[.664, 1.423]	.050	1.036	[.758, 1.416]
Block 2a	Static-99R	.558	1.080	[.883, 1.321]	.016	.984	[.765, 1.265]	.013	1.023	[.693, 1.511]	.308	1.095	[.796, 1.505]
	SAPROF-SO	4.708	.965*	[.934, .997]	3.111	.964	[.926, 1.004]	.825	.971	[.912, 1.034]	1.522	.968	[.919, 1.019]
Block 2b	Static-99R	.471	1.072	[.879, 1.309]	.072	.966	[.753, 1.240]	.006	1.015	[.689, 1.495]	.214	1.078	[.785, 1.480]
	Resilience	5.475	.933*	[.880, .989]	2.493	.942	[.875, 1.014]	.872	.946	[.843, 1.062]	1.160	.950	[.864, 1.043]
Block 2c	Static-99R	.211	1.048	[.859, 1.278]	.000	.998	[.779, 1.279]	.032	1.035	[.709, 1.512]	.339	1.097	[.803, 1.499]
	Adaptive sexuality	1.596	.937	[.847, 1.037]	6.202	.837*	[.728, .963]	1.738	.866	[.698, 1.073]	2.339	.874	[.735, 1.039]
Block 2d	Static-99R	.179	1.043	[.857, 1.270]	.400	.923	[.719, 1.184]	.043	.959	[.649, 1.418]	.071	1.044	[.760, 1.433]
	Prosocial connection & reward	1.857	.939	[.857, 1.028]	.001	.999	[.892, 1.118]	.121	1.032	[.863, 1.234]	.060	.982	[.848, 1.137]

*Note. R*^*2*^(Nagelkerke) Any Revocation Block 1 = .000, Block 2a = .039, Block 2b = .045, Block 2c = .013, Block 2d = .015, Sexually Related Revocation Block 1 = .004, Block 2a = .035, Block 2b = .029, Block 2c = .068, Block 2d = .004, Sexual Offense Revocation Block 1 = .000, Block 2a = .013, Block 2b = .014, Block 2c = .029, Block 2d = .002, Any Offense Revocation Block 1 = .001, Block 2a = .020, Block 2b = .015, Block 2c = .031, Block 2d = .001.

*p < .05.

Our Hosmer-Lemeshow test results suggested that the models involving both Static-99R and total SAPROF-SO scores were a poor fit for the data pertaining to sexually related revocations (χ2 = 25.776, *p* = .001) and revocations for sexual offenses (χ2 = 15.647, *p* = .048). To test whether this was due to non-linear effects, quadratic and cubic terms were added in the regression analyses, this involved stepwise addition of SAPROF-SO squared (SAPROF-SO^*2*^) and SAPROF-SO cubed (SAPROF-SO^*3*^) values. Following addition of both the SAPROF-SO^*2*^ (Block 3) and SAPROF-SO^*3*^ (Block 4) values, the model fit improved in both cases (see Table 7). Further, for sexually related revocations, both the models including SAPROF-SO^*2*^ alone and SAPROF-SO^*2*^ together with SAPROF-SO^*3*^ demonstrated statistical significance (*χ2* (3, *N* = 170) = 8.896, *p* = .031 and *χ2* (4, *N* = 170) = 11.381, *p* = .023 respectively). Indeed, after adding the SAPROF-SO^*3*^ value, the model correctly classified 81.7% of cases and explained up to 10.6% of the variance in sexually related revocation outcomes. In line with these findings, Table 8 suggests a non-linear relationship between mean SAPROF-SO scores such that scores over 2.00 are associated with better outcomes but below that threshold SAPROF-SO is not related to revocation.

**Table 7.** Quadratic Hierarchical Logistical Regression Predicting Likelihood of Sexual Offense and Sexually Related Revocation for Total Sample (*N* = 170) During the 24 Month Follow-Up Period.

		Sexually related revocation				Sexual offense revocation			
Scale		Wald (*df* = 1)	Exp(B)	95% CI	Hosmer and lemeshow χ2 (sig.)	Wald (*df* = 1)	Exp(B)	95% CI	Hosmer and lemeshow χ2 (sig.)
Block 1	Static-99R	.424	.922	[.723, 1.177]	.305 (.998)	.021	.972	[.664, 1.423]	3.686 (.596)
Block 2	Static-99R	.016	.984	[.765, 1.265]		.013	1.023	[.693, 1.511]	
	SAPROF-SO	3.111	.964	[.926, 1.004]	25.776 (.001)	.825	.971	[.912, 1.034]	15.647 (.048)
Block 3	Static-99R	.052	.971	[.756, 1.248)		.006	1.015	[.689, 1.496)	
	SAPROF-SO	2.617	1.171	[.967, 1.417]		.111	1.043	[.812, 1.340]	
	SAPROF-SO^2^	4.030	.995*	[.990, 1.000]	8.646 (.373)	.333	.998	[.992, 1.004]	9.815 (.278)
Block 4	Static-99R	.143	.952	[.737, 1.229)		.020	.971	[.646, 1.459)	
	SAPROF-SO	.667	.825	[.519, 1.309]		2.762	.585	[.310, 1.101]	
	SAPROF-SO^2^	1.383	1.017	[.989, 1.047]		2.817	1.036	[.994, 1.081]	
	SAPROF-SO^3^	2.121	1.000	[.999, 1.000]	10.114 (.257)	2.754	.999	[.999, 1.000]	9.623 (.292)

*Note. R*^*2*^(Nagelkerke) Sexually Related Revocation Block 1 = .004, Block 2 = .035, Block 3 = .083, Block 4 = .106, Sexual Offense Revocation Block 1 = .000, Block 2 = .013, Block 3 = .019, Block 4 = .076.

*p < .05.

**Table 8.** Percentage of Participants Revoked for Sexual Offenses or Sexually Related Supervised Release Rules Breaches by Mean SAPROF-SO Score.

Mean SAPROF-SO score	*N* participants	Sexually related revocations (%)	Sexual offense revocations (%)
1.00 or less	46	12 (41.02)	4 (16.78)
1.01–2.00	76	17 (41.59)	6 (14.20)
2.01 or more	48	2 (7.41)	1 (3.70)

Our analyses also indicated that the Adaptive Sexuality subscale (*χ2* (1, *N* = 170) = 6.202, *p* = .013) and the SAPROF-SO^*2*^ score (*χ2* (1, *N* = 170) = 4.030, *p* = .045) made a statistically significant unique contribution to predicting the likelihood of sexually related revocations. Inverting the associated odds ratios suggested that for each unit increase in Adaptive Sexuality Subscale score there was a 19.5% decrease in the likelihood of sexually related revocation and for each unit increase in SAPROF-SO^*2*^ score the likelihood decreased by .5%. In addition, the SAPROF-SO total (*χ2* (1, *N* = 170) = 4.708, *p* = .030) and the Resilience subscale (*χ2* (1, *N* = 170) = 5.475, *p* = .019) scores made statistically significant unique contributions to predicting the likelihood of any revocation. The results suggested that single unit increases in each of these variables were associated with decreases in revocation rates of 4% and 7%, respectively.

## Discussion

The present study adds to the process of validating the SAPROF-SO; a relatively new instrument for the assessment of factors that protect against sexual violence risk. The results of our interrater reliability analyses were consistent with Nolan and colleagues’ (2023) findings that the SAPROF-SO can be reliably coded from the administrative records of adult males who have been convicted of sexual offenses. In turn, our ROC results showed that SAPROF-SO scores can validly predict supervised release success in a US context involving adult males identified as being of significantly higher risk of sexual recidivism than those in the samples used in the Nolan et al. study, or in any other validation studies to date. Specifically, the ROC results indicated that the more SAPROF-SO protective factors identified for such an individual at the time of supervised release, the less likely they would be revoked and returned to the treatment center due to breaches of supervised release conditions. Further, higher total SAPROF-SO scores were also found to discriminate between individuals whose supervised release was revoked due to sexual offending or associated behaviors.

Our findings also suggested that higher protective factors scores on two of the SAPROF-SO subscales were significantly related to supervised release success. The Resilience subscale was found to validly discriminate adult males who were revoked for any reason (most commonly breaches of supervised release rules) from those who were not, while the Adaptive Sexuality subscale discriminated between adult males who were and were not revoked for sexual offending and/or behaviors aligned with prior sexual offenses. The Adaptive Sexuality subscale findings are particularly noteworthy, as this subscale is one of the characteristics of the SAPROF-SO that most distinguishes it from other protective factors (or risk) assessment instruments. The Adaptive Sexuality subscale includes items related to sexual self-regulation (regulation of sexual impulses and evidence of a normative sex drive), prosocial sexual interests (interest in and arousal to consenting adult sex), prosocial sexual identity (acceptance of a prosocial adult sexual orientation), and intimate relationship (romantic, physical relationship of good quality and stability). Although the SAPROF also considers the presence and quality of intimate relationships, the SAPROF-SO item is arguably more focused, as it includes consideration of sexual behavior within such relationships. Further, neither the SAPROF, nor any other known measure of protective factors, includes the other Adaptive Sexuality subscale items. Although some dynamic risk assessment tools include items that could be seen as the inverse of those on the Adaptive Sexuality subscale (e.g., STABLE-2007 sex as coping and deviant sexual preferences items, VRS-SO deviant sexual preference, sexual compulsivity and sexually deviant lifestyle items), as argued earlier, the absence of a risk item does not necessarily equate to the presence of its protective alternative.

The current study failed to reveal an association between SAPROF-SO Prosocial Connection & Reward subscale scores and revocation. Given that prosocial connection (including involvement in work, pro-social leisure activities, friendships and social support networks) has repeatedly been identified as statistically protective of recidivist offending (see Worling & Langton, 2022), this was somewhat unexpected. However, considering the context of the current study, it is possibly not surprising. Our SAPROF-SO scores were derived from data collected immediately prior to participants’ release from a secure treatment center in the US. At that time, almost all participants were involved in a therapeutic work program and most were housed, and shared social support and leisure pursuits with others who were also awaiting supervised release (and therefore likely motivated to reduce involvement in anti-social activity). However, once on supervised release, opportunities for such social or occupational activity tended to be very limited, particularly during the first 12 months. Therefore, it is likely that these adult male’s pre-release SAPROF-SO Prosocial Connection & Reward scores bore little relation to the scores they would have achieved during the initial 12–24 months post supervised release.

The lack of effect associated with the Prosocial Connection & Reward subscale highlights the externally oriented nature of many of the protective factors in it. While in some ways easier to influence or modify than the more internally focused factors of the Resilience and Adaptive Sexuality subscales, factors associated with the construct of Prosocial Connection & Reward are arguably hardest to maintain without external support to do so. As stated by Lussier and McCuish (2016), in the US at least, societal attitudes and post-release conditions may actually limit the likelihood that adult males who have been convicted of sexual offenses will ever be able to form the types of social connections that have been shown to reduce recidivism. In turn, the current results may suggest that supervised release outcomes could be improved if treatment centers (and relevant legislation) were more open to supporting opportunities for associated activity earlier during adult male’s supervised release experiences. Of course, it is possible that one of the factors that currently prevents this is the fact that pre-release assessments that focus primarily on relatively limited, unchangeable static risk factors make it difficult for administrators to know who will both benefit most from this, and be least likely to abuse it. As identified in the current study, less than half of the adult males in our sample had been administered the main dynamic risk assessment instrument (VRS-SO) used by the treatment center as part of their pre-release planning.

Although the magnitude of the effects observed in the current study fell in the small to moderate range, the SAPROF-SO clearly out-performed (and demonstrated incremental predictive ability in relation to) the Static-99R, which showed no ability to predict the likelihood of supervised release revocation for any reason. Given that the Static-99R is one of the most commonly used measures of recidivism risk amongst individuals who have been convicted of sexual offenses, and was also most commonly used in the context in which the current research was undertaken, this finding may appear surprising. It is notable that in the present study the Static-99R showed little predictive value in relation to both sexual recidivism (which it is designed to predict) and revocation for any reason (which it was not designed to predict). This may simply reflect sampling error since the confidence intervals for the relevant AUC statistics were wide. However, it is likely that the relatively homogenous (above average) static risk profile and the fact that the criminal history items would typically reflect behaviors two decades in the past may also have reduced the Static-99R’s ability to discriminate recidivism. Notably, despite having more statistical power, a recent study following individuals after discharge or dismissal from civil commitment as SVPs in Wisconsin also found that Static-99R scores “did not discriminate [sexual] recidivists from non-recidivists”, suggesting that more than sampling error is involved (Mundt et al., in press, p.1).

Logistic regression analyses examining the incremental contribution to prediction of sexual offending by the SAPROF-SO over and above the Static-99R did not yield statistically significant results. Rather the SAPROF-SO showed incremental prediction in relation to broader outcome criteria that included sexual offending but also included other reasons for revocation. This likely reflects lack of statistical power for analyses of sexual recidivism consequent on the small number of sexual recidivists. The question of the incremental contribution of the SAPROF-SO to the prediction of sexual offending should be addressed with more statistical power in future studies. It is unclear how reliable the apparent non-linear effects identified in the logistic regression analyses will prove to be. The non-linear effects are not large and future samples with a much greater N will be required to replicate them and, if replicated, to define their contours. In the meantime, the practical implication would be that evaluators should only infer a protective effect relative to revocation for SAPROF-SO scores over 2.0.

### Implications

In addition to adding to the SAPROF-SO validation process, the current research has a number of implications in terms of the prediction, management and prevention of sexual offense risk when planning and administering release from criminal justice contexts. In particular, our findings highlight the challenges associated with relying on static risk assessment instruments when planning post-release supervision of adult males who have been convicted of sexual offenses. As discussed earlier, the fact that these instruments only measure factors that cannot be improved means that their ability to inform associated intervention is very limited, and generally restriction focused. In turn, they may bias criminal justice agencies towards actions that actually reduce the potential for protective factors (such as prosocial connections) to be maintained, and inadvertently contribute to increased risk over the longer term.

Similarly, our findings suggest that in populations of adult males who demonstrate relatively homogenous, above average static risk profiles, the ability of static instruments to validly predict sexual offense recidivism may be very limited. As such, dependence on these instruments during judicial decision making may mean that some adult males who are unlikely to re-offend, will continue to be detained. However, it may also mean that adult males who are at risk of recidivism may be prematurely discharged. As discussed earlier, decisions regarding unconditional discharge commonly rely on the findings of actuarial measures, of which static risk assessment instruments are the most commonly used. In contrast, to obtain supervised release, individuals must demonstrate treatment progress. In the absence of consistent use of measures of dynamic risk and protective factors, it is harder to reliably show evidence of sustained change. In turn, as demonstrated in our results, it may actually be easier for adult males of above average recidivism risk to meet the statutory criteria for discharge than for supervised release.

Together, these findings highlight the need to increase and/or ensure consistent use of dynamic assessment instruments in criminal justice contexts. Further, although we were unable to estimate the extent to which assessment of dynamic risk could have improved prediction of supervised release revocation, our results clearly showed the benefits of including measures of dynamic protective factors. In addition, they confirmed that assessment of factors that protect against sexual recidivism requires not only measurement of factors that have been shown to protect against violence generally, but also factors specifically related to adaptive sexuality.

### Limitations and Future Directions

The current study had a number of limitations. Although the retrospective nature of the design enabled relatively quick and efficient data collection, it also meant that data integrity could not be ensured. As discussed, the archival information that was used to score the SAPROF-SO did not always include information relevant to each SAPROF-SO item, with information relating to three of the Prosocial Connection & Reward subscale items being amongst the most commonly missing. As such, it is possible that associated omissions undermined our ability to accurately assess the predictive ability of this subscale and draw associated conclusions. It is also possible that the information used to score other items was not complete or fully representative of each man’s experience. As such, future research into comparable populations would benefit from a design involving a wider range of data collection methods (e.g., combining archival and self-report methods).

The current research was also limited by the possibility that the Prosocial Connection & Reward subscale scores at time of release were not representative of participants’ experiences during supervised release. As such, use of longitudinal, repeated measures designs is advocated. Further, it would be helpful to follow participants for a longer period than 24 months. Relevant literature advocates a minimum three to five years follow-up period for studies investigating sexual offense recidivism outcomes (Collaborative Outcome Data Committee, 2007). Unfortunately, due to the time limited nature of most of the supervised release arrangements under consideration (and associated data collection practices), we were unable to achieve this. Going forward, this could be enabled by ensuring that research and administrative consent processes include the option of collecting ongoing follow-up data even after participants are no longer subject to supervised release conditions.

It is important to emphasize that this study used a sample of individuals who were determined to have a predisposing mental condition, prior conviction(s) for sexually violent offenses, and a Static-99R result placing them in the Above Average to Well Above Average risk level. Further, the supervised release conditions for individuals committed under SVP laws are significantly more restrictive than those on community supervision generally (e.g., parole and probation). This likely accounts for the low base-rate for those living with these supervised release conditions (Ambroziak et al., 2023). Thus, while the results are likely generalizable to other SVP and similarly selected samples, they will not be generalizable to individuals in routine community supervision scenarios. It would be helpful for future research studies to both investigate the predictive validity of the SAPROF-SO for individuals who were unconditionally discharged from their SVP commitment as well as those who are on routine community supervision conditions.

Future studies should also aim to sample more participants. This is particularly important given the very low base rates of sexual recidivism generally, and the even lower levels likely to occur under the highly restrictive conditions of supervised release. Although there is good reason to consider revocations for any breach of supervised release conditions or for sexual offense analogous behaviors as relevant to the prediction of sexual recidivism, neither is likely to be as relevant to that outcome (or comparable with other research) as actual sexual offense recidivism measures.

Finally, further work is needed to examine the incremental validity of the SAPROF-SO against measures of dynamic risk assessment. In addition, for the purpose of generalization across a broader range of contexts, it would also be helpful to validate the SAPROF-SO on populations of individuals with similar static risk levels who have not been subjected to civil commitment or associated prolonged, highly restricted sentences and conditions.

## Conclusion

In summary, our findings add to the process of validating the SAPROF-SO. They showed that scores on this instrument can be reliably collected from administrative data and used to predict supervised release success in a sample of adult males identified as being of significantly higher risk of sexual recidivism than those sampled in other validation studies to date. In addition, our research demonstrated the utility of the SAPROF-SO Adaptive Sexuality subscale in the prediction of sexually related revocations. Further, although the magnitude of associated effects fell in the small to moderate range, the SAPROF-SO clearly out-performed the Static-99R which, despite being one of the most commonly used sexual offense recidivism risk assessment instruments, showed no ability to predict the likelihood of supervised release revocation for any reason. These findings have a number of implications for predicting and managing sexual offense risk when planning and administering release from criminal justice contexts, including the potential benefits of consistently assessing both dynamic risk and protective factors.
